# Oral Health Service Utilisation Behaviour and Its Influencing Factors Among Older Adults: An Empirical Study Based on the Composite Health Behaviour Model

**DOI:** 10.3290/j.ohpd.c_2822

**Published:** 2026-09-29

**Authors:** Xiaoyuan Pan, Shuwen Su, Jianli Li, Leyi Han, Xinyue Chen

**Affiliations:** a Xiaoyuan Pan* BDS, Foshan Stomatological Hospital, School of Medicine, Foshan University, Foshan 528225, China; Postgraduate student, Department of Preventive Dentistry, School and Hospital of Stomatology, Guangdong Engineering Research Center of Oral Restoration and Reconstruction and Guangzhou Key Laboratory of Basic and Applied Research of Oral Regenerative Medicine, Guangzhou Medical University, Guangzhou, 510182, China; responsible for the design and concept, conducted the study, and co-wrote and reviewed the manuscript; b Shuwen Su* Associate Professor, Foshan Stomatological Hospital, School of Medicine, Foshan University, Foshan 528225, China; responsible for the design and concept, conducted the study, and co-wrote and reviewed the manuscript; c Jianli Li BDS, Foshan Stomatological Hospital, School of Medicine, Foshan University, Foshan 528225, China; analysed the data and results, responsible for visualisation, and co-wrote the manuscript; d Leyi Han Undergraduate student, Foshan Stomatological Hospital, School of Medicine, Foshan University, Foshan 528225, China; performed the investigation and co-wrote the manuscript; e Xinyue Chen Undergraduate student, Foshan Stomatological Hospital, School of Medicine, Foshan University, Foshan 528225, China; performed the investigation and co-wrote the manuscript *Equal contribution.

**Keywords:** geriatric oral health, health belief model, health services utilisation, structural equation modelling, theory of planned behaviour

## Abstract

**Background:**

China’s aging population faces significant unmet oral health care needs, with delayed service utilisation exacerbating disease burden. Currently, there is a lack of empirical research applying composite health models and analysing pathway relationships among influencing factors in this population.

**Methods:**

A cross-sectional study was conducted from July to September 2023 among 356 older adults in Foshan and Guangzhou using a theory-driven questionnaire. Group comparisons used independent *t* tests/analysis of variance for sociodemographic variables. Structural equation modelling (SEM) with maximum likelihood estimation was employed to validate pathways.

**Results:**

Significant differences were observed: sex and age affected individual model variables; education, residence location, and economic status influenced multiple variables; and living arrangement showed no significant effects. Only 34.04% of older adults proactively sought oral health knowledge, while 65.03% did not receive regular dental checkups. Just 51.84% sought treatment (scaling, fillings, extractions, dentures, implants). When acute oral pain disrupted daily life, 25.15% still delayed medical care. SEM revealed that behavioural intention/control had the strongest total effect on utilisation behaviour (β = 0.593) followed by perceived benefits (β = 0.404), subjective norms exerted only indirect effects (β = 0.216), and both subjective norms (β = 0.365) and perceived benefits (β = 0.282) directly influenced behavioural intention and control.

**Conclusion:**

Oral health service utilisation among older adults remains passive. The present authors’ model identifies three key modifiable factors: behavioural intention and control, perceived benefits, and subjective norms. Strategies to enhance service utilisation should focus on strengthening social support and modifying unrealistic health perceptions.

Oral health service utilisation is defined as ‘the actual amount of dental services received by residents, reflecting both service provision and institutional efficiency’.^3^ Regular utilisation enables early detection and management of oral diseases through preventive care, effectively maintaining oral health while reducing long-term treatment frequency and costs. As China’s population ages rapidly, with the number of adults aged ≥ 60 years increasing by 5.44% from 2010 to 2020 and reaching 264.02 million (18.7% of the population) in 2020,^22^ older adults face disproportionately poor oral health. The Fourth National Oral Health Epidemiological Survey of China revealed alarming statistics among those aged 65 to 74 years: 98% had decayed/missing/filled teeth (DMFT), 90.3% exhibited dental calculus, and only 9.3% maintained periodontal health.^24^ This underscores an urgent need to address oral health disparities in China’s aging population.

These findings indicate suboptimal oral health behaviours among older Chinese adults, where behavioural factors represent critical modifiable targets. Influences on older adults’ behaviour are multifaceted, yet few domestic studies apply established behavioural theories to investigate oral health service utilisation. Existing research inadequately explores psychosocial determinants and lacks theoretical foundations for interventions. Behavioural theories, beyond categorising and predicting oral health habits, guide interventions from individual to population levels. As emphasised by the UK National Institute for Health and Care Excellence (NICE), ‘theory-based behaviour change interventions demonstrate greater efficacy than atheoretical approaches’.^1^ Globally, preventive dentistry increasingly employs behavioural theories to enhance self-care and treatment adherence, evidenced by the 11th European Workshop on Periodontology recommending psychological methods for plaque control in periodontal management.^21^ Consequently, theoretically grounded identification of influencing factors and pathways represents the vanguard of oral health interventions.^4^


Among behavioural frameworks, the health belief model (HBM) and theory of planned behaviour (TPB) are most established. This study therefore integrates the HBM and TPB to develop a composite model specific to older adults’ oral health service utilisation, aiming to elucidate behavioural mechanisms and inform targeted interventions.

## Materials and methods

### Theoretical Framework and Composite Model Construction

There is a growing consensus that theory-based behavioural research offers greater explanatory and predictive reliability.^18^ However, existing theoretical models remain insufficient to fully account for the variance observed in oral health behaviours. Consequently, future model development may need to consider further refinements to existing frameworks to enhance their predictive capacity.^20^ Some scholars have suggested that integrating two or more theoretical models to provide novel analytical perspectives on behaviour change may represent an important direction for the evolution of health behaviour theory.^6,27
^ Nevertheless, the combined application of models remains context-specific: given the substantial heterogeneity across different health behaviours, even identical constructs may exert differential effects depending on the behavioural domain. To date, integrated theoretical models have been developed primarily for specific behaviours rather than as universal frameworks.

Drawing upon the classic HBM and TPB frameworks, this study developed an integrated and optimised composite model tailored to the characteristics of older adults, with the aim of better explaining oral health service utilisation behaviour in this population. The HBM and TPB are conceptually complementary: the former emphasises perceived threats, benefits, and barriers as drivers of health behaviour, whereas the latter highlights the roles of subjective norms and behavioural intention.^7^ Given the conceptual overlap between the two models, the present authors streamlined similar dimensions and proposed a hypothesised composite model. The detailed model integration process, including construct merging and questionnaire development, is described in the methodological preprint.^15^ In brief, the composite model comprises six latent variables: perceived benefit (PBE), perceived barrier (PBA), perceived threat (PT), subjective norm (SN), behavioural intention and control (BIC), and behaviour (B). Their definitions are presented in Table 1, and the hypothesised pathways are illustrated in Fig 1.

**Table 1 Table1:** Newly constructed composite model building block variable definitions

Variable	Definition
PBE	Subjective evaluation held by the older adults regarding the potential positive outcomes of engaging in oral health service utilisation behaviour
PBA	Subjective assessment of the difficulties potentially encountered during the process of oral health service utilisation behaviour
PT	Subjective assessment of the risk of disease and its consequences
SN	Individual’s subjective perception of social pressure concerning whether or not to adopt oral health service utilisation behaviour
BIC	Capability and autonomy to perform oral health service utilisation behaviour
B	Oral health service utilisation behaviour


**Fig 1 Fig1:**
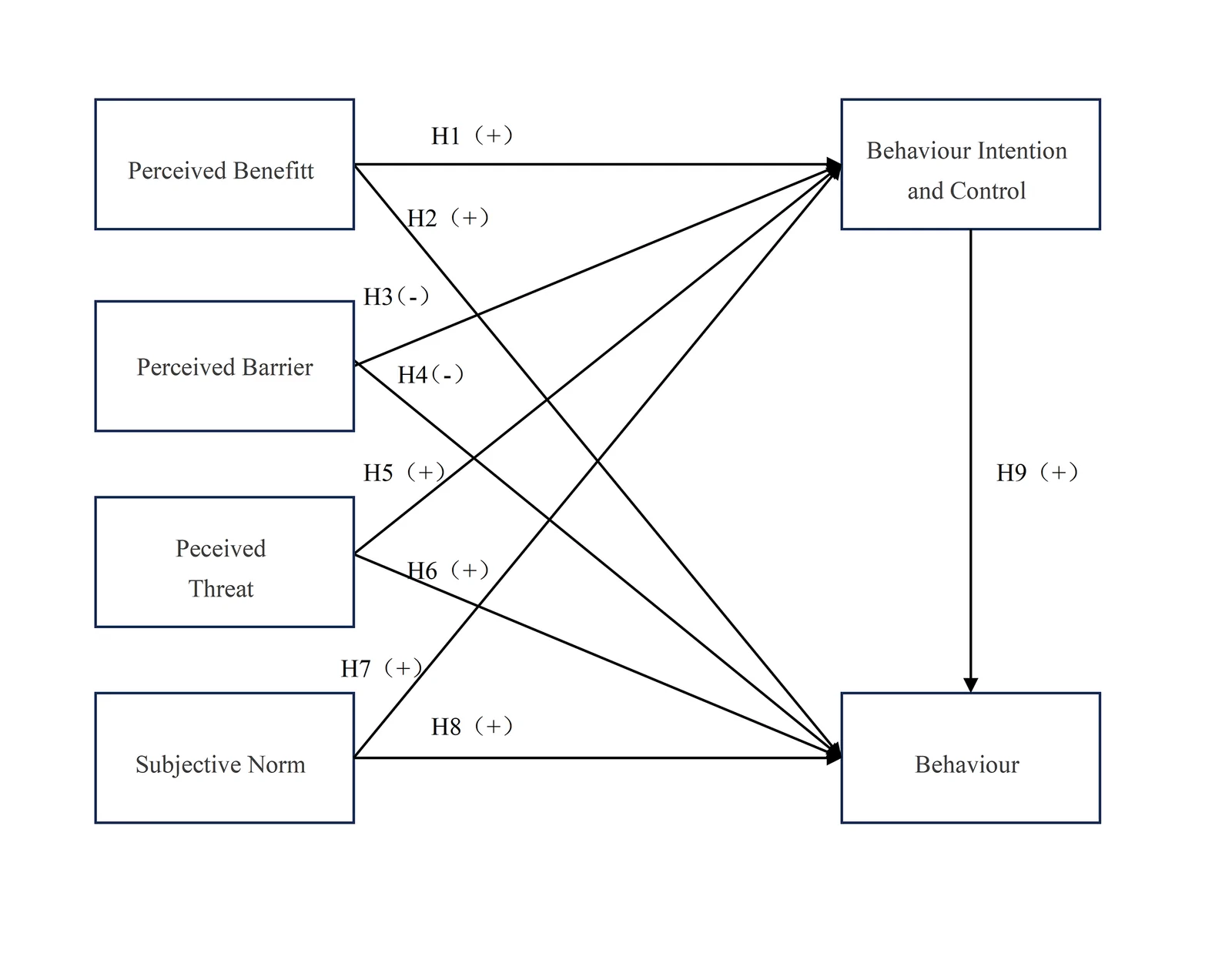
Path relationship assumptions between the newly constructed composite model components. H1, PBE has a positive effect on BIC; H2, PBE has a positive effect on B; H3, PBA has a negative effect on BIC; H4, PBA has a negative effect on B; H5, PT has a positive effect on BIC; H6, PT has a positive effect on B; H7, SN has a positive effect on BIC; H8, SN has a positive effect on B; H9, BIC has a positive effect on B.

### Research Methods

Based on the hypothesised composite model described above, a structured questionnaire was developed through a systematic process of construct merging, item generation, expert review, and pilot testing, as detailed in the methodological preprint.^15^ In brief, the final questionnaire comprised 26 items distributed across six dimensions corresponding to the model variables: PBE, PBA, PT, SN, BIC, and B. All items were rated on a 5-point Likert scale ranging from ‘strongly disagree’ (1) to ‘strongly agree’ (5), with higher scores indicating more positive perceptions or behaviours. The questionnaire demonstrated acceptable psychometric properties: exploratory factor analysis extracted six principal components accounting for 62.616% of the cumulative variance, with factor loadings ranging from 0.517 to 0.817, and the overall Cronbach α was 0.864.

Sociodemographic characteristics were assessed using a structured set of single-choice questions. Participants were asked to select their sex (male/female), age (60 to 69, 70 to 79, ≥ 80 years), education level (primary school, junior high school, high school/secondary specialised school, college/associate degree, master’ s degree or above), place of residence (rural/urban), economic status (very affluent, relatively comfortable, financially sufficient, some financial difficulty, severe financial difficulty), and living arrangements (living alone, living with spouse, living with children, other).

#### Questionnaire design

A questionnaire was self-developed by integrating the HBM and the TPB. The present study referenced established scales and drew upon previous research findings to formulate items for each dimension. Items for B, PBE, PBA, and PT were developed based on the research by Nakazono et al^14^ and Xiang et al,^26^ items for SN were adapted from the work of Liu,^28^ and items for BIC were developed referencing studies by Ju^8^ and Sun.^19^


The questionnaire was specifically designed considering the behavioural and psychological characteristics of the older adults. It underwent refinement through expert review and a pilot pre-survey before finalisation. The final questionnaire comprised 26 items across six dimensions: B, PBE, PBA, PT, SN, and BIC. Given the potentially limited comprehension abilities of older adult respondents, all items utilised a 5-point Likert scale, ranging from ‘strongly disagree’ (scored 1) to ‘strongly agree’ (scored 5), with higher scores indicating more positive responses. Exploratory factor analysis (EFA) extracted six principal components, accounting for a cumulative variance contribution rate of 62.616%. Factor loadings for all items ranged from 0.517 to 0.817. The overall Cronbach α coefficient for the scale was 0.864, indicating good reliability and validity.^15^


#### Data collection

This cross-sectional study was conducted from July to September 2023. Participants were older adults recruited by convenience sampling from five residential communities in Foshan and Guangzhou, China. Recruitment was facilitated through community bulletin boards, local neighbourhood committee announcements, and on-site promotion during free oral health talk events. Eligible individuals were those aged ≥ 60 years, cognitively able to complete the questionnaire (either independently or with assistance), and willing to provide written informed consent.

Questionnaires were administered using paper forms and electronic surveys (through the Wenjuanxing platform), with the mode of completion determined by participant preference and mobility. Participants completed the questionnaire either in community health service centres or in their own homes, while trained researchers provided standardised verbal guidance. All research assistants were oral health care professionals who used a standardised script to explain the study purpose without leading responses, and performed real-time checks on questionnaire completeness. Before participation, all individuals were informed of the study purpose, the voluntary nature of their participation, and the confidentiality of their responses. The study protocol was approved by the Medical Ethics Committee of Foshan University (ethics approval no. 2022001), and written informed consent was obtained from all participants.

Two independent researchers applied predefined exclusion criteria: patterned responses (e.g., straight-lining), contradictory answers within the same dimension, and logical inconsistencies (e.g., reporting no dental visits but detailing specific treatments). A total of 356 older adults were recruited and 352 questionnaires were returned. After excluding 26 incomplete or invalid responses, 326 valid questionnaires were retained for analysis. Given the 26 measurement items, the final sample size (n = 326) satisfied the common ‘10 times rule’ (minimum 10 cases per measured item) for structural equation modelling (SEM).^13,23,25
^


#### Statistical analysis

Data processing and analysis were performed using SPSS (version 19.0; IBM, Armonk, NY, USA) and AMOS software (version 24.0; IBM). Descriptive statistics (frequencies, percentages) were used to characterise the sociodemographic profile of the surveyed older adult participants and describe the current status of their oral health service utilisation behavior. SEM was employed to assess the goodness-of-fit of the integrated model of older adults’ oral health service utilisation behaviour. Effect analysis and path analysis were conducted on the integrated model within the SEM framework.

## Results

### Analysis of Sociodemographic Characteristics

This study identified the basic characteristics of the surveyed participants across dimensions including sex, age, education level, place of residence, economic status, and living arrangements. Questionnaire results showed that participants were aged ≥60 years, and there were 132 men and 194 women. The proportion of participants with education levels above high school or secondary specialised education was relatively low. Only 11% of the older adults respondents self-reported experiencing family economic difficulties. A majority (82.3%) of the older adults lived with their spouse or children. The sample covered younger-old (60 to 69 years), middle-old (70 to 79 years), and older-old (≥ 80 years) age groups. Specific details are presented in Table 2.

**Table 2 Table2:** Sociodemographic characteristics of older adults interviewed (n = 326)

Variable	Group	Frequency (n)	Percentage (%)
Sex	Male	132	40.50
	Female	194	59.50
Age, y	60–69	172	52.80
	70–79	109	33.40
	≥ 80	45	13.80
Education level	Primary school	112	34.40
	Junior high school	80	24.50
	High school/secondary specialised school	68	20.90
	College/associate degree	54	16.60
	Master’s degree +	12	3.70
Place of residence	Rural	148	45.40
	Urban	178	54.60
Economic status	Very affluent	10	3.10
	Relatively comfortable	96	29.40
	Financially sufficient	184	56.40
	Some financial difficulty	32	9.80
	Severe financial difficulty	4	1.20
Living arrangements	Living alone	48	14.70
	Living with spouse	142	43.60
	Living with children	126	38.70
	Other	10	3.10


Univariate analysis was employed to examine the variables of sex, age, education level, etc., within the personal background of the older adult participants, aiming to explore differences in psychological and behavioural manifestations of oral health service utilisation across different group characteristics. As these variables could not be incorporated into the SEM, univariate analysis was conducted using SPSS (version 19.0).

Each of the four background variables, namely age, education level, economic status, and place of residence, was divided into at least three groups. Therefore, one-way analysis of variance (ANOVA) was used to test for between-group differences in each variable across the questionnaire dimensions. Sex and place of residence were divided into only two groups, so independent samples *t* tests were used for analysis.

The results indicated that significant differences were found for sex and age in individual aspects of the model variables and also across multiple aspects for participants grouped by education level, place of residence, and economic status, but no significant differences were found among participants grouped by living arrangements (*P* > 0.05). Detailed data are presented in Table 3.

**Table 3 Table3:** Behavioural and psychological variables of oral health service utilisation among the older adults grouped by sociodemographic factors and psychological variables

Factor	Group	B, mean ± SD	Statistic^§^	PBE, mean ± SD	Statistic^§^	PBA, mean ± SD	Statistic^§^	SN,mean ± SD	Statistic^§^	PT, mean ± SD	Statistic^§^	BIC, mean ± SD	Statistic^§^
Sex	Male (n = 132)	2.77 ± 1.08	*t* = −1.778	4.06 ± 0.73	*t *= −0.600	3.08 ± 0.73	*t *= −0.290	3.50 ± 0.95	*t* = −2.594*	3.39 ± 0.71	*t* = −0.620	3.40 ± 0.80	*t* = −2.089*
	Female (n = 194)	2.99 ± 1.05		4.11 ± 0.71		3.11 ± 0.75		3.77 ± 0.89		3.44 ± 0.76		3.61 ± 0.80	
Age (y)	60–69 (n = 172)	2.98 ± 1.03	*F* = 3.510*	4.15 ± 0.71	*F *= 1.370	3.08 ± 0.77	F = 1.337	3.65 ± 0.89	*F *= 0.067	3.51 ± 0.69	*F* = 3.557*	3.56 ± 0.80	*F* = 2.147
	70–79 (n = 109)	2.91 ± 1.06		4.05 ± 0.67		3.06 ± 0.69		3.65 ± 0.92		3.28 ± 0.78		3.58 ± 0.73	
	≥ 80 (n = 45)	2.56 ± 1.19		3.97 ± 0.83		3.27 ± 0.75		3.70 ± 1.09		3.39 ± 0.76		3.28 ± 0.94	
Education level	Primary (n = 112)	2.92 ± 1.06	*F* = 8.083***	3.98 ± 0.71	*F* = 3.184*	3.22 ± 0.63	*F *= 3.276*	3.57 ± 0.93	*F* = 0.756	3.34 ± 0.66	*F* = 3.716***	3.37 ± 0.81	*F* = 4.993*
	Junior high school (n = 80)	2.92 ± 1.06		4.01 ± 0.78		3.14 ± 0.76		3.69 ± 0.89		3.35 ± 0.83		3.48 ± 0.83	
	High school/secon dary (n = 68)	3.00 ± 1.09		4.23 ± 0.62		2.83 ± 0.78		3.67 ± 1.05		3.42 ± 0.74		3.62 ± 0.79	
	College + (n = 54)	3.23 ± 0.99		4.16 ± 0.76		3.09 ± 0.71		3.71 ± 0.82		3.50 ± 0.71		3.60 ± 0.70	
	Master’s + (n = 68)	4.00 ± 0.62		4.58 ± 0.35		3.25 ± 1.19		4.00 ± 0.80		4.15 ± 0.50		4.37 ± 0.50	
Residence	Rural (n = 148)	2.64 ± 1.01	*t* = −4.526***	3.98 ± 0.73	*t* = −2.497*	3.21±0.65	*t* = 2.361*	3.58 ± 0.89	*t* = −1.399	3.36 ± 0.72	*t* = −1.191	3.36 ± 0.80	*t* = −3.407**
	Urban (n = 178)	3.12 ± 1.07		4.18 ± 0.69		3.01 ± 0.81		3.72 ± 0.95		3.46 ± 0.75		3.66 ± 0.78	
Economic status	Very affluent (n = 10)	3.23 ± 1.31	*F* = 2.988*	4.07 ± 0.72	*F* = 3.277*	3.24± 1.09	*F* = 1.426*	3.90 ± 0.90	*F* = 0.478	3.77 ± 0.79	*F* = 0.837	3.84 ± 0.79	*F* = 1.677
	Comfortable (n = 96)	2.99 ± 1.06		4.17 ± 0.60		3.01 ± 0.74		3.69 ± 0.88		3.40 ± 0.67		3.56 ± 0.78	
	Sufficient (n = 184)	2.93 ± 1.04		4.13 ± 0.67		3.10 ± 0.75		3.65 ± 0.94		3.43 ± 0.77		3.54 ± 0.79	
	Some difficulty (n = 32)	2.46 ± 1.05		3.69 ± 1.06		3.36 ± 0.59		3.53 ± 0.98		3.29 ± 0.78		3.33 ± 0.86	
	Severe difficulty (n = 4)	2.08 ± 1.32		3.75 ± 1.17		3.10 ± 0.58		3.33 ± 1.25		3.46±0.44		2.80 ± 1.12	
Living arrangement	Alone (n = 48)	2.82 ± 1.17	*F* = 1.193	3.98 ± 0.85	*F* = 2.010	3.03 ± 0.67	*F* = 1.757	3.65 ± 0.87	*F* = 1.038	3.41 ± 0.82	*F* = 1.267	3.33± 0.94	*F* = 1.706
	With spouse (n = 142)	2.99 ± 0.96		4.19 ± 0.64		3.18 ± 0.75		3.71 ± 0.86		3.50 ± 0.69		3.62 ± 0.73	
	With children (n = 126)	2.80 ± 1.12		4.01 ± 0.75		3.01 ± 0.76		3.57 ± 1.01		3.34 ± 0.71		3.50 ± 0.79	
	Other (n = 10)	3.23 ± 1.40		4.30 ± 0.55		3.36 ± 0.69		4.03 ± 1.02		3.23 ± 1.24		3.44 ± 1.18	
**P* < 0.05; ***P* < 0.01; ****P* < 0.001; ^§^ *t*-value for two-group comparisons; *F*-value for three or more groups.													

### Descriptive Analysis of Oral Health Service Utilisation Behaviour

In the behavioural questionnaire, only 34.04% of older adult participants reported proactively learning about oral health knowledge. A substantial majority (65.03%) indicated they did not proactively seek regular oral checkups, and only 51.84% would proactively seek dental treatment (e.g., cleaning, fillings, extractions, dentures, or implants). When acute oral pain affected daily life, 25.15% still reported not seeking timely medical care (Table 4). These empirical findings indicate low oral health awareness among older adults; both the awareness and behaviour of proactively and regularly seeking oral health services were at alarmingly low levels, representing a key intervention point. They also show inefficient health service utilisation; current oral health service utilisation among older adults is predominantly passive, with low efficiency. Pain-driven utilisation (seeking care only when experiencing discomfort) is widespread.

**Table 4 Table4:** Current status of oral health service utilisation behaviours among older people (n=326)

Item	Strongly disagree, n (%)	Disagree, n (%)	Uncertain, n (%)	Agree, n (%)	Strongly agree, n (%)
Proactively seek oral health knowledge	46 (14.11)	106 (32.52)	63 (19.33)	91 (27.91)	20 (6.13)
Schedule regular dental checkups	59 (18.10)	96 (29.45)	57 (17.48)	89 (27.30)	25 (7.67)
Seek preventive/therapeutic dental services (e.g. scaling, filling, extraction)	48 (14.72)	70 (21.47)	39 (11.96)	129 (39.57)	40 (12.27)
Visit dental practitioners promptly for acute oral pain affecting everyday life	22 (6.75)	25 (7.67)	35 (10.74)	149 (45.71)	95 (28.14)


### Validation and Analysis of SEM for Older Adults’ Oral Health Service Utilisation Behaviour

#### Model fit verification

Based on the theoretically hypothesised model of older adults’ oral health service utilisation behaviour established previously, SEM was constructed using AMOS (version 24.0). In this hypothesised model, four variables, namely PBE, PBA, PT, and SN, were specified as exogenous latent variables, while BIC and B were specified as endogenous latent variables. This framework constituted the SEM for older adults’ oral health service utilisation behaviour, schematically represented in Fig 2.

**Fig 2 Fig2:**
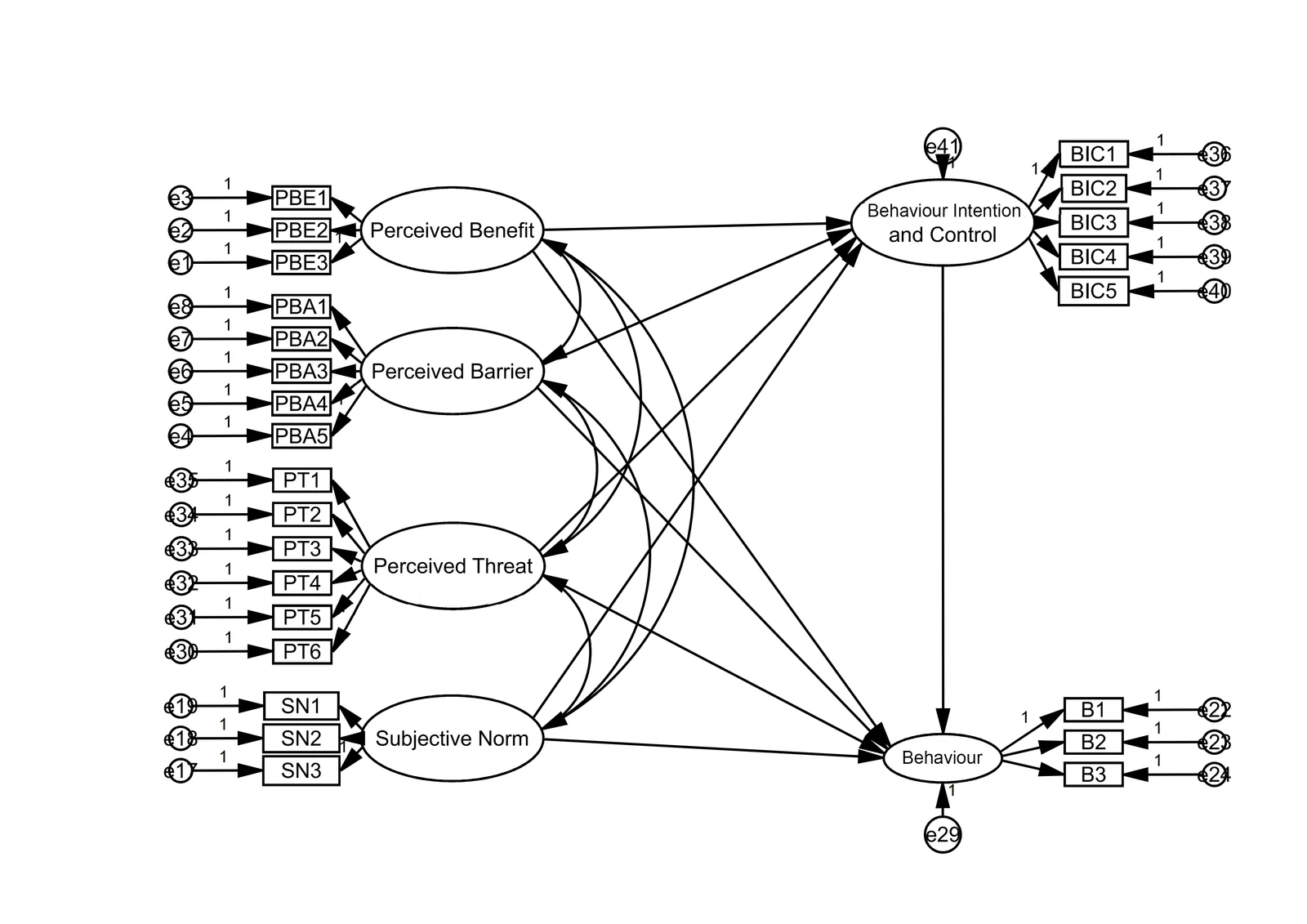
Hypothetical modelling diagram.

Prior to conducting path analysis, the reliability and validity of the hypothesised model must be established through model fit assessment; that is, by importing data into the constructed model for estimation. Prevailing methodological consensus holds that overall Goodness-of-Fit Indices provide a feasible means to evaluate the model’s fit, verifying the extent to which the hypothesised model aligns with the observed data. Consequently, this study employed a comprehensive set of overall fit indices to assess model adequacy. The global fit of the structural equation model was evaluated across three categories: Absolute Fit Indices, Incremental Fit Indices, and Parsimonious Fit Indices. The formal questionnaire data were imported into AMOS 24.0 for processing. Absolute Fit Indices are presented in Table 5, Incremental Fit Indices in Table 6, and Parsimonious Fit Indices in Table 7. Synthesising these results, the model demonstrated reasonable fit, indicating that the hypothesised model for this study is acceptable.

**Table 5 Table5:** Absolute Fit Indices for SEM

Index	Abbreviation	Criterion	Value	Evaluation
Chi-square/degrees of freedom	Χ^2^/df	< 3	2.476	Reasonable fit
Root mean residual	RMR	0.05–0.08	0.069	Reasonable fit
Root mean square error of approximation	RMSEA	0.05–0.08	0.067	Reasonable fit
Goodness-of-Fit Index	GFI	> 0.8	0.859	Reasonable fit
Adjusted GFI	AGFI	0.8–0.9	0.823	Reasonable fit


**Table 6 Table6:** Incremental Fit Indices for SEM

Index	Abbreviation	Criterion	Value	Evaluation
Normed Fit Index	NFI	> 0.8	0.824	Reasonable fit
Relative Fit Index	RFI	> 0.8	0.797	Marginal fit*
Incremental Fit Index	IFI	> 0.8	0.887	Reasonable fit
Tucker-Lewis Index	TLI	> 0.8	0.868	Reasonable fit
Comparative Fit Index	CFI	> 0.8	0.886	Reasonable fit
*RFI value (0.797) is marginally below the threshold but acceptable given other indices.				

**Table 7 Table7:** Parsimonious Fit Indices for SEM

Index	Abbreviation	Criterion	Value	Evaluation
Parsimonious GFI	PGFI	> 0.5	0.687	Reasonable fit
Parsimonious Normed Fit Index	PNFI	> 0.5	0.712	Reasonable fit
Parsimonious Comparative Fit Index	PCFI	> 0.5	0.768	Reasonable fit


#### Analysis of research path hypothesis verification

A core function of constructing SEM is to conduct an in-depth analysis of the path dependency relationships among multiple complex variables, aiming to reveal potential patterns of interaction between them. Therefore, following the model fit assessment, it is necessary to further test the original research hypotheses. Path analysis, as an effective tool within SEM suitable for handling continuous variable data, builds upon the analysis of correlations between variables to explore and infer potential causal chains.

This study employed the maximum likelihood (ML) method for hypothesis testing of path causal relationships. In testing path coefficients, a research hypothesis is accepted only if the standardised coefficient is significantly different from zero and *P* < 0.05. Otherwise, the null hypothesis is retained.

Analysis of Table 8 reveals that PBE and SN had significant effects on BIC (*P* < 0.001), PT and PBA did not have significant effects on BIC (*P* > 0.05), BIC and PBE had significant effects on B (*P* < 0.001), and PT, PBA, and SN did not have significant direct effects on B (*P* > 0.05). Consequently, the hypothesis validation results are presented in Table 9. As shown, hypotheses H1, H2, H7, and H9 were supported in this study, whereas the remaining hypotheses were not.

**Table 8 Table8:** Path coefficients of the SEM of oral health service utilisation behaviour of older people

Path	Unstandardised coefficient	Standard error	Critical ratio	*P* value
BIC ← PBE	0.276	0.067	4.140	***
BIC ← PT	0.187	0.074	2.512	0.012
BIC ← SN	0.337	0.065	5.203	***
BIC ← PBA	−0.070	0.064	−1.089	0.276
B ← BIC	0.834	0.129	6.461	***
B ← PT	−0.159	0.102	−1.554	0.120
B ← PBA	−0.034	0.090	−0.382	0.702
B ← PBE	0.330	0.097	3.396	***
B ← SN	0.008	0.089	0.089	0.929
**P* < 0.05; ***P* < 0.01; ****P* < 0.001.				

**Table 9 Table9:** Hypothesis validation results

Hypothesis	Statement	Result
H1	PBE → BIC (+)	Supported
H2	PBE → B (+)	Supported
H3	PBA → BIC (-)	Not supported
H4	PBA → B (-)	Not supported
H5	PT → BIC (+)	Not supported
H6	PT → B (+)	Not supported
H7	SN → BIC (+)	Supported
H8	SN → B (+)	Not supported
H9	BIC → B (+)	Supported


The magnitude of the path relationships, i.e., the influence between latent variables, is expressed through the path coefficients. Extracting the standardised path coefficients and reconstructing the model diagram based on the supported hypotheses (H1, H2, H7, and H9), the overall path relationships influencing older adults’ oral health service utilisation behaviour are depicted in Fig 3.

**Fig 3 Fig3:**
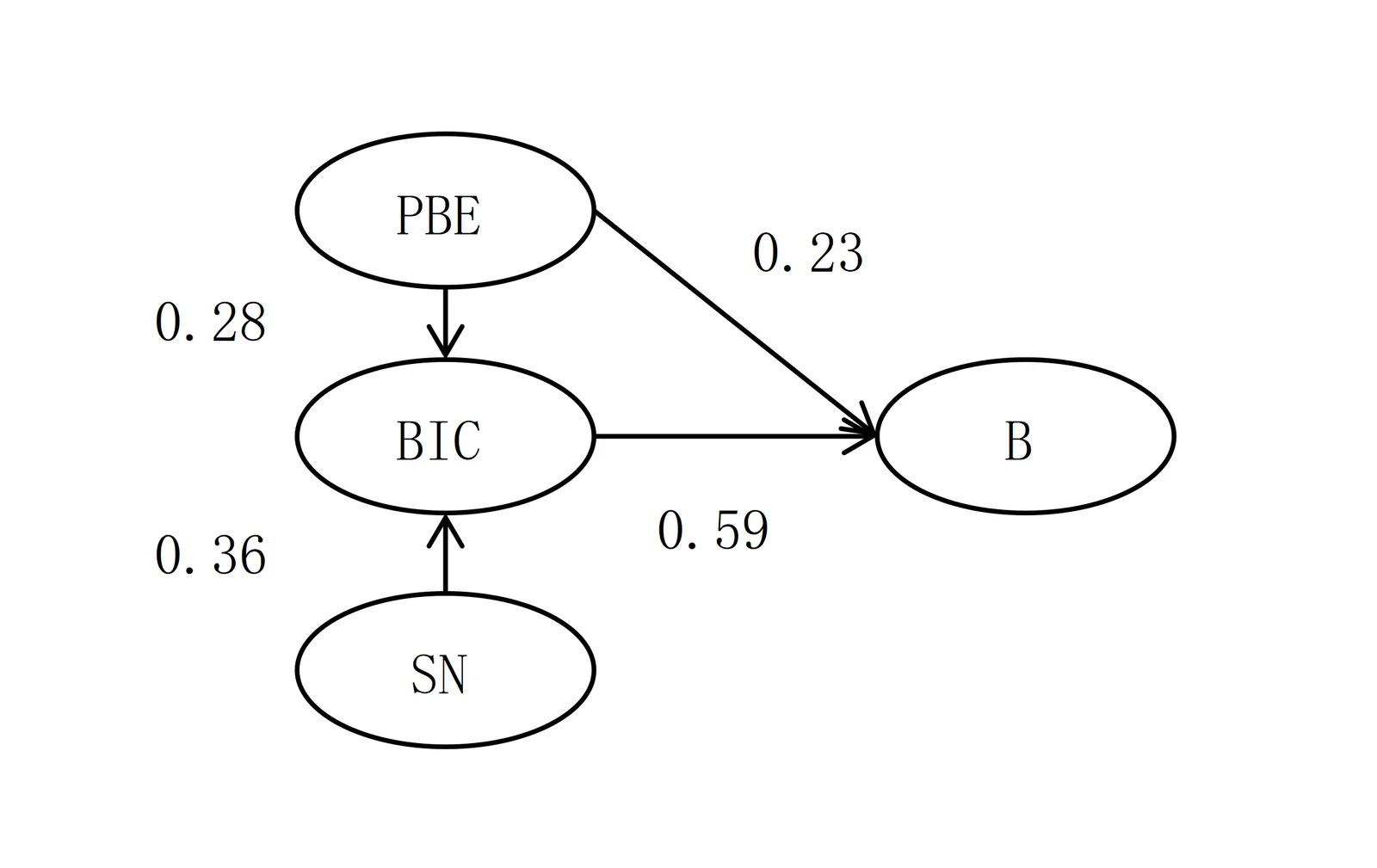
General pathways of behavioural mechanisms for oral health service utilisation by older people. Arrows indicate significant paths and values represent standardised coefficients. The total effect of BIC on B is 0.593.

As illustrated in Fig 3, the influence of the four latent variables (PBE, SN, BIC, and B) on older adults’ oral health service utilisation behaviour manifests through three primary pathways. The variable BIC has a direct positive effect on B, and this path exhibits the largest total effect (β = 0.593); PBE can both directly positively influence B and indirectly positively influence it through its effect on the BIC variable; and SN can only indirectly positively influence B through the mediating role of the BIC variable.

#### Effect analysis

The effects within the model can be categorised into direct, indirect, and composite total effects. The direct effect is represented by the standardised path coefficient between variables, the indirect effect is calculated as the product of the standardised path coefficients along a mediated pathway, and the total effect is the sum of the direct and indirect effects. Based on the magnitude of total effects, the primary factors influencing older adults’ oral health service utilisation behaviour were ranked in descending order as BIC, PBE, and SN. Specific values are presented in Table 10.

**Table 10 Table10:** Analysis of model effects

Dependent variable	Independent variable	Direct effect	Indirect effect	Total effect
B	BIC	0.593	NA	0.593
	PBE	0.239	0.165	0.404
	SN	NA	0.216	0.216
BIC	SN	0.365	NA	0.365
	PBE	0.282	NA	0.282
NA, not applicable.				

The empirical data demonstrate that BIC exerted the strongest influence on B (total effect 0.593), PBE had the second strongest influence (total effect 0.404), and SN had the smallest influence, with no direct effect and only an indirect effect (indirect effect 0.216; total effect 0.216). Furthermore, regarding the factors influencing BIC, both SN and PBE exerted only direct effects (0.365 and 0.282, respectively), with no indirect pathways.

## Discussion

### Principal Findings: Low Utilisation Despite High Need

The Fourth National Oral Health Epidemiological Survey^24^ revealed concerning oral health status among the group aged 65 to 74 years, suggesting a ‘high need’ for oral health services. However, the present authors’ study on the current status of oral health service utilisation among older adults in China identified a ‘low utilisation’ phenomenon. This contradiction between high need and low utilisation underscores the critical necessity for interventions targeting older adults’ oral health service utilisation behaviours. Thus, there is an urgent need to intervene on influencing factors to improve the adoption rate of oral health utilisation behaviours and promote older adults’ oral health.

This ‘high need–low utilisation’ paradox is not unique to China, however. A multi-country analysis using World Health Organization Strategic Advisory Group of Experts on Immunization (WHO SAGE) data reported that the prevalence of self-reported unmet needs for oral health services among adults aged ≥ 50 years was 60% in China, 80% in Ghana, and 62% in India, with lower education levels consistently associated with higher unmet needs across all three countries.^9^ Even in high-income settings with relatively high overall attendance, significant intranational disparities persist. Kovács et al,^11^ analysing longitudinal data from 20,803 older adults across 14 European countries, reported that while annual dental care attendance was highest in Scandinavian countries (85.7%), the difference in service utilisation between socioeconomic groups was expanding over time regarding income level and residential area. This suggests that high national average utilisation rates may mask growing internal inequalities, implying that improving overall access alone is insufficient without targeted efforts to reach disadvantaged subpopulations.

These international comparisons collectively underscore that the ‘high need–low utilisation’ contradiction is a global geriatric oral health challenge, transcending national boundaries and health care systems. Therefore, interventions targeting modifiable behavioural and psychosocial determinants, rather than merely expanding service supply, are urgently needed to improve oral health service utilisation among older populations worldwide.

### Key Influencing Pathways and Their Implications

The structural equation model revealed three significant pathways (*P* < 0.001) through which the composite model variables influenced oral health service utilisation behaviour among older adults. The first of these was a direct positive path from BIC to B. When older adults possess stronger behavioural intention and perceived control, their capability and autonomy to perform the behaviour are enhanced. These individuals are also more likely to believe in the effectiveness and benefits of utilising oral health services, thereby increasing the likelihood of performing the behaviour. This finding not only aligns with the fundamental assumptions of TPB but is also supported by multiple empirical studies.^2,12,16
^


The influence path of PBE resembles findings by Kiyak et al.^10^ PBE can both directly positively influence B and indirectly influence it through BIC acting as a mediating variable. This conclusion bridges constructs from distinct models, namely PBE from classic HBM and BIC from traditional TPB, establishing a novel influence pathway for older adults’ oral health service utilisation behaviour.

SN did not establish a direct link with B in the composite model. Rather, it exerted an indirect positive effect on older adults’ oral health service utilisation behaviour through the mediating role of BIC. This pathway of SN influencing behaviour solely through indirect means is consistent with the classical TPB.

### Practical Implications for Intervention

The newly conceptualised BIC demonstrated the most significant influence on B among all factors (total effect 0.593). Research by Gamma et al^5^ confirms the high correlation between behavioural intention and behaviour. Furthermore, studies indicate that perceived behavioural control is a major predictor of both oral health behavioural intention and actual behaviour, explaining 34% of behavioural variance.^17^ This supports the role of BIC as a crucial predictor of older adults’ oral health service utilisation behaviour. PBE ranked second in influence (total effect 0.404), followed by SN (no direct effect, only indirect effect = 0.216). In social life, perceived benefit is a powerful driver of individual behaviour. Compared to external directives, elderly individuals are more inclined to perform behaviours they perceive as personally beneficial. Furthermore, regarding the factors influencing BIC, both SN and PBE exerted only direct effects (0.365 and 0.282, respectively). As a vulnerable group, older adults often consult family, friends, or health care professionals when encountering unfamiliar matters. These opinions significantly impact their behavioural intention and control (SN had a larger direct effect on BIC [0.365] than PBE [0.282]). Concurrently with this social influence, older adults often receive tangible assistance from these sources (e.g., family members accompanying them to dental visits). This assistance not only boosts behavioural intention but also directly enhances the resources and capability required to perform oral health service utilisation behaviours.

Based on the identified key dimensions, PBE, SN, and BIC, the present authors propose the following multi-level intervention strategies:

1.Enhancing PBE: On an individual/family level, this would involve recognising the decline in self-directed learning capacity among older adults. Family members should actively accompany elders, share relevant oral health knowledge, watch educational television programmes together, consult oral health care books with them, and answer their questions. On a societal level, mainstream media should prioritise oral health promotion. Health care institutions could establish specialised medical teams for community outreach programmes, enhancing interactivity during educational sessions and bridging the gap with the public through community-based initiatives. Government bodies should also optimise the allocation of oral health human resources and reasonably distribute oral health care resources to improve service experiences, thereby enhancing PBE.2.Leveraging SN: On an individual level, it is essential for older adults to actively listen to and consider advice from relatives, friends, or health care personnel regarding oral health. On a family level, families can foster a home environment that values oral health, encourage elders to develop good oral hygiene habits, and regularly remind and accompany them to utilise oral health services. On a societal level, similar to the family approach, society-wide oral health education should be promoted to cultivate a broader societal atmosphere that prioritises oral health.3.Strengthening BIC: This dimension, encompassing classical concepts of behavioural intention and perceived behavioural control, is the most influential in the model and serves as a proximal determinant of behaviour. Recommendations under PBE and SN will inherently contribute to improving BIC levels. Additionally, as BIC includes the capability to perform the behaviour, this can be bolstered by external support. Practical support would include family members accompanying elders to dental appointments and hospitals improving service accessibility and convenience. Genuine care and companionship for elders may be the most effective catalyst for promoting health care–seeking behavior.

## Conclusion

Under the analytical framework integrating the HBM and TPB, this study employed empirical data to investigate the key influencing factors and underlying mechanisms of the ‘low utilisation’ phenomenon in oral health service utilisation behaviour among older adults.

Within the composite model, the identified influencing factors operate through distinct pathways. The variable BIC exerts a direct positive effect on B, demonstrating the largest total effect; PBE positively influences older adults’ oral health service utilisation behaviour both directly and indirectly by affecting BIC; and SN can only positively influence older adults’ oral health service utilisation behaviour indirectly, mediated through the BIC variable. Thus, it is recommended to implement strategies that focus on enhancing social support, addressing cognitive misconceptions, and strengthening the expectations and support from family, friends, and health care professionals. These interventions can effectively target SN, BIC, and PBE, ultimately promoting proactive utilisation of oral health services among older adults.

Collectively, this study provides a scientific foundation and effective strategies for improving oral health status among older adults, contributing novel perspectives and insights to advance the process of healthy aging.

This study was funded by grants from the National Natural Science Foundation of China (grant number 72204046), Guangdong Philosophy and Social Science Foundation (grant number GD24XGL003), Foshan Science and Technology Innovation Project (grant number 2320001007325), and Guangdong Basic and Applied Basic Research Foundation (grant number 2019A1515111102.

## References
